# Avatrombopag Reduces Platelet Transfusion Requirement in Thrombocytopenia Subsequent to Antineoplastic Therapies in Haematological Patients: The Experience of a Tertiary Centre

**DOI:** 10.3390/jcm15052044

**Published:** 2026-03-07

**Authors:** Aser Alonso-Carballo, Marta López, María Jiménez, Sandra Pérez, Lucía García-Mañó, Jose María Sánchez, Leyre Bento, Andrés Novo, Albert Pérez, Carmen Ballester, Antonio Gutiérrez, Guiomar Puget, Bernat Galmés, Antonio Palomero, Antonia Sampol, Mariana Canaro

**Affiliations:** 1 Haematology and Haemotherapy Service, Son Espases University Hospital, 07120 Palma, Spain; 2 Hospital Pharmacy Service, Son Espases University Hospital, 07120 Palma, Spain

**Keywords:** antineoplastic therapy-induced thrombocytopenia, platelet transfusion, thrombopoietin receptor agonist, Avatrombopag, haematological malignancy, bleeding

## Abstract

**Background/Objectives:** Thrombocytopenia subsequent to antineoplastic therapies leads to bleeding complications, treatment delay or de-intensification, and platelet transfusion requirement. Evidence suggests that thrombopoietin receptor agonists (TPO-RAs) can restore platelet counts in this scenario. Avatrombopag (AVA) is an oral TPO-RA whose efficacy in treating thrombocytopenia in haematological malignancy has been barely addressed. We aimed to evaluate AVA’s efficacy in improving platelet recovery and reducing transfusion requirement in haematological patients with thrombocytopenia. **Methods**: In this retrospective observational study, haematological patients who developed thrombocytopenia persisting for ≥3 weeks and were treated with AVA between November 2023 and December 2024 were recruited. **Results**: Twenty-three patients were recruited. Nineteen (82.6%) responded to AVA, most within the first 4 weeks: 10 (43.5%) and 9 (39.1%) achieved platelet counts ≥ 30 × 10^9^/L (partial response) and ≥100 × 10^9^/L (complete response), respectively. Response was always maintained for 30 days after AVA withdrawal. Transfusions were significantly fewer than in the previous period: 0 (0–8) vs. 11 (2–15), median (interquartile range [IQR]), *p* = 0.007. Once on treatment, 13 (56.5%) patients no longer required transfusion. No patient delayed or de-intensified chemotherapy. No safety concerns were reported. **Conclusions**: AVA shows promise in safely reducing thrombocytopenia-associated transfusion needs in haematological malignancy.

## 1. Introduction

Antineoplastic therapy-induced thrombocytopenia is a common complication of cancer therapy, affecting up to one-third of patients with solid tumours and nearly half of those with haematological malignancies, depending on cancer type and regimen [1,2]. According to the Common Terminology Criteria for Adverse Events (CTCAE) v5.0, antineoplastic therapy-induced thrombocytopenia is considered to occur when platelet count falls below 100 × 10^9^/L [3]. Patients with thrombocytopenia often require platelet transfusions and are at significant risk of severe bleeding [1,4]. Thrombocytopenia increases morbidity, prolongs hospitalisation and, importantly, disrupts anticancer treatment through dose delays or reductions, lowering relative dose intensity (RDI) and potentially compromising survival outcomes [5]. As anticipated, platelet transfusion is currently the gold standard for managing antineoplastic therapy-induced thrombocytopenia, particularly in severe cases [6]. This procedure is costly, resource-intensive, and carries risks such as alloimmunisation [1,7]. Despite the prevalence of thrombocytopenia, no specific therapy beyond platelet transfusion has been approved for its prevention or management.

Thrombopoietin receptor agonists (TPO-RAs) are a class of drugs that mimic the action of thrombopoietin to stimulate platelet production. TPO-RAs are currently indicated to treat immune thrombocytopenia (ITP), aplastic anaemia, and liver disease-related thrombocytopenia [8,9,10]. Experience with TPO-RAs in the antineoplastic therapy-induced thrombocytopenia scenario is limited and predominantly focused on solid tumours (reviewed by Al-Samkari) [5]. Nevertheless, a recent expert review stated that the current body of evidence strongly supports the use of TPO-RAs for managing persistent thrombocytopenia [11].

Avatrombopag (AVA) is the most recently developed TPO-RA. AVA is administered orally and does not have known interactions with foods or drinks. For this reason, its use has been increasing steadily in recent years [12]. The literature addressing the use of AVA for managing antineoplastic therapy-induced thrombocytopenia is encouraging but scarce, with reports focusing predominantly on solid tumours (reviewed by Lozano et al.) [13]. Regarding thrombocytopenia subsequent to intensive chemotherapy, one of the pioneering studies suggested that patients who most benefit from AVA are those with persistent thrombocytopenia rather than nadir thrombocytopenia [14]. Other series described promising results, always in patients with solid tumours [15,16,17]. Interestingly, another study found that AVA was as effective as autologous platelet transfusion to resolve thrombocytopenia [18]. In the field of haematological malignancy, studies regarding the role of AVA have been conducted in paediatric acute lymphoblastic leukaemia, post-chemotherapy aplasia, and post-allogeneic haematopoietic stem cell transplantation (allo-HSCT), often consisting of case reports or case series with a limited number of patients [19,20,21,22,23]. The role of TPO-RAs in restoring platelet counts after CAR-T cell therapy has been even less studied and has focused on eltrombopag and romiplostim [24]. In any case, the aforementioned studies suggest that AVA presents a favourable efficacy and safety profile for managing antineoplastic therapy-induced thrombocytopenia. With this background, we sought to share the experience of our centre with AVA used for managing haematological patients with persistent thrombocytopenia secondary to intensive chemotherapy, allo-HSCT-related and chimeric antigen receptor T (CAR-T) cell therapy-related procedures. Response to AVA in terms of platelet count recovery, subsequent avoidance of transfusion, maintenance of scheduled chemotherapy, and safety outcomes is reported.

## 2. Methods

### 2.1. Patients, Design and Aims of the Study

**Figure 1:**
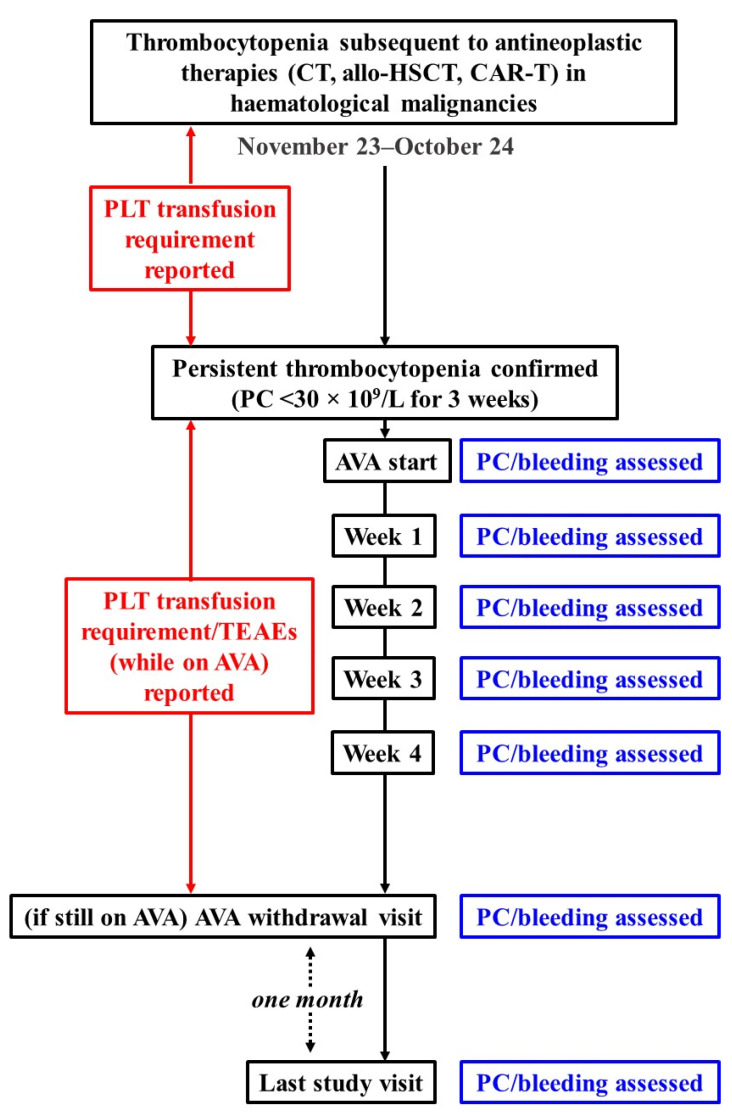
Flowchart diagram of the study. Procedures, assessments, period of patient recruitment, and follow-up end date are indicated.

A retrospective, observational, single-centre study was designed by researchers of the Son Espases University Hospital (Palma de Mallorca, Balearic Islands, Spain). The different steps are shown in the flowchart diagram (Figure 1). The primary aims were the comparison of platelet transfusion requirement in oncohaematological patients diagnosed with antineoplastic therapy-induced thrombocytopenia in the periods before and after starting therapy with AVA, response to AVA in terms of platelet count recovery, and assessment of safety and tolerability while treatment was being administered.

### 2.2. Inclusion and Exclusion Criteria

Inclusion criteria were age over 18 years, a diagnosis of haematological cancer regardless of cell lineage, onset of thrombocytopenia secondary to either intensive chemotherapy (CIT) or any procedure associated with allo-HSCT or CAR-T cell therapy, persistence of thrombocytopenia, i.e., platelet counts < 30 × 10^9^/L, for 3 weeks, and a decision to initiate AVA therapy after this period to restore platelet counts. Exclusion criteria were a diagnosis of other disorders known to influence platelet counts, a recent history of cardiovascular disease or arterial or venous thrombosis, and previous exposure to any TPO-RA.

### 2.3. Platelet Transfusion Requirement

Platelet transfusion was carried out whenever patients had either platelet counts < 10 × 10^9^/L, or platelet counts < 50 × 10^9^/L and active bleeding.

### 2.4. Treatment, Response Criteria and Safety Assessment

AVA therapy was always initiated at a daily dose of 20 mg. Doses would be escalated to 40 mg/day in the event that platelet counts remained <30 × 10^9^/L after 14 days. Response was defined as partial (PR) or complete (CR) when platelet counts increased to ≥30 × 10^9^/L or ≥100 × 10^9^/L, respectively, with no platelet transfusion requirement for at least 7 days. AVA therapy was suspended in the event that platelet counts did not achieve levels ≥ 30 × 10^9^/L at 14 days after the first dose was administered. Platelet counts were monitored for a minimum of one month after the first dose of AVA was administered. When patients responded to AVA, the treatment was suspended: 14 days after platelet count recovery if PR was achieved; 7 days after platelet count recovery if CR was achieved; in patients on chemotherapy, at cycle end, provided that PR or CR had been achieved even although the period of time since response was reported was shorter than 14 or 7 days, respectively.

In order to assess the safety profile of the treatment, treatment-emergent adverse events (TEAEs) were reported while patients remained on AVA therapy. Platelet transfusion requirement was assessed before and after the start of the treatment. Leukocyte counts (TLC) and circulating levels of haemoglobin (Hb), alanine aminotransferase (ALT), aspartate aminotransferase (AST), gamma-glutamyl transferase (GGT) and alkaline phosphatase (ALP) were systematically assessed every 7 days during the first 4 weeks after the start of AVA treatment.

### 2.5. Follow-Up

For each patient, the last visit was defined as that attended one month after the definitive suspension of the AVA treatment. Thus, the study was considered to have ended one month after the latest AVA suspension.

### 2.6. Ethics

The study was approved by the Institutional Review Board of Son Espases University Hospital (CI-1096-25; approval date 22 October 2025) and conducted in accordance with the Declaration of Helsinki.

### 2.7. Statistical Analysis

Continuous variables were represented by median (interquartile range [IQR]), and qualitative variables were summarised as numbers and percentages. The two-tailed Wilcoxon test and the two-tailed Mann–Whitney U test were used to compare paired and unpaired quantitative variables, respectively. The two-tailed Fisher’s exact test and the chi-square test were used to compare dichotomous or polytomous variables, respectively. The two-tailed Spearman’s rho test was used to assess correlations between quantitative and qualitative variables. The Kruskal–Wallis test was used to compare the evolution of laboratory variables during the first 4 weeks of treatment. In case of statistical significance, the post hoc Dunn’s test was subsequently applied. Statistical significance was established at *p* < 0.05, and GraphPad Prism 5 software was used for calculations.

## 3. Results

### 3.1. Baseline Features of the Cohort

Patient demographic and baseline characteristics are outlined in Table 1. Twenty-three patients were recruited between November 2023 and October 2024. Among them, 61% were male, and 48% of the total were diagnosed with lymphoproliferative disorders. Although more than half of the patients received intensive chemotherapy, up to 35% underwent allogeneic bone marrow transplantation. The median (IQR) follow-up period from the onset of antineoplastic therapy-induced thrombocytopenia to study end was 86 (76–123) days.

### 3.2. Time from Onset of Thrombocytopenia to Initiation of AVA Therapy

**Figure 2:**
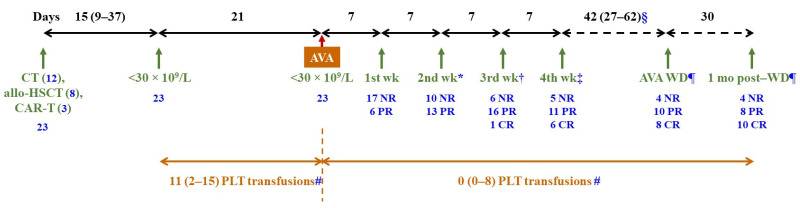
Graphical summary of the efficacy outcomes of AVA in the overall cohort. The median (IQR) time between the start of antineoplastic therapies and onset of thrombocytopenia is shown. The number of platelet transfusions before and after AVA treatment initiation, and response to AVA at selected times (number of patients in NR, PR or CR), are also indicated. * One patient suspended treatment at this time point due to no response. † Three patients suspended treatment at this time point, due to no response or prolonged response in two and one cases, respectively, and one patient who had achieved PR was lost to follow-up due to transfer to another hospital. ‡ Nine patients suspended treatment at this time point, due to no response or prolonged response in one and eight cases, respectively. § The median (IQR) time elapsed between the 28th day of treatment and AVA withdrawal was calculated considering only the nine patients who remained on AVA treatment after 4 weeks. ¶ Responses of the patients who suspended AVA in the first 28 days of treatment are also included. # Transfusion was administered with bleeding symptoms and/or PC < 10 × 10^9^/L.

**Figure 3:**
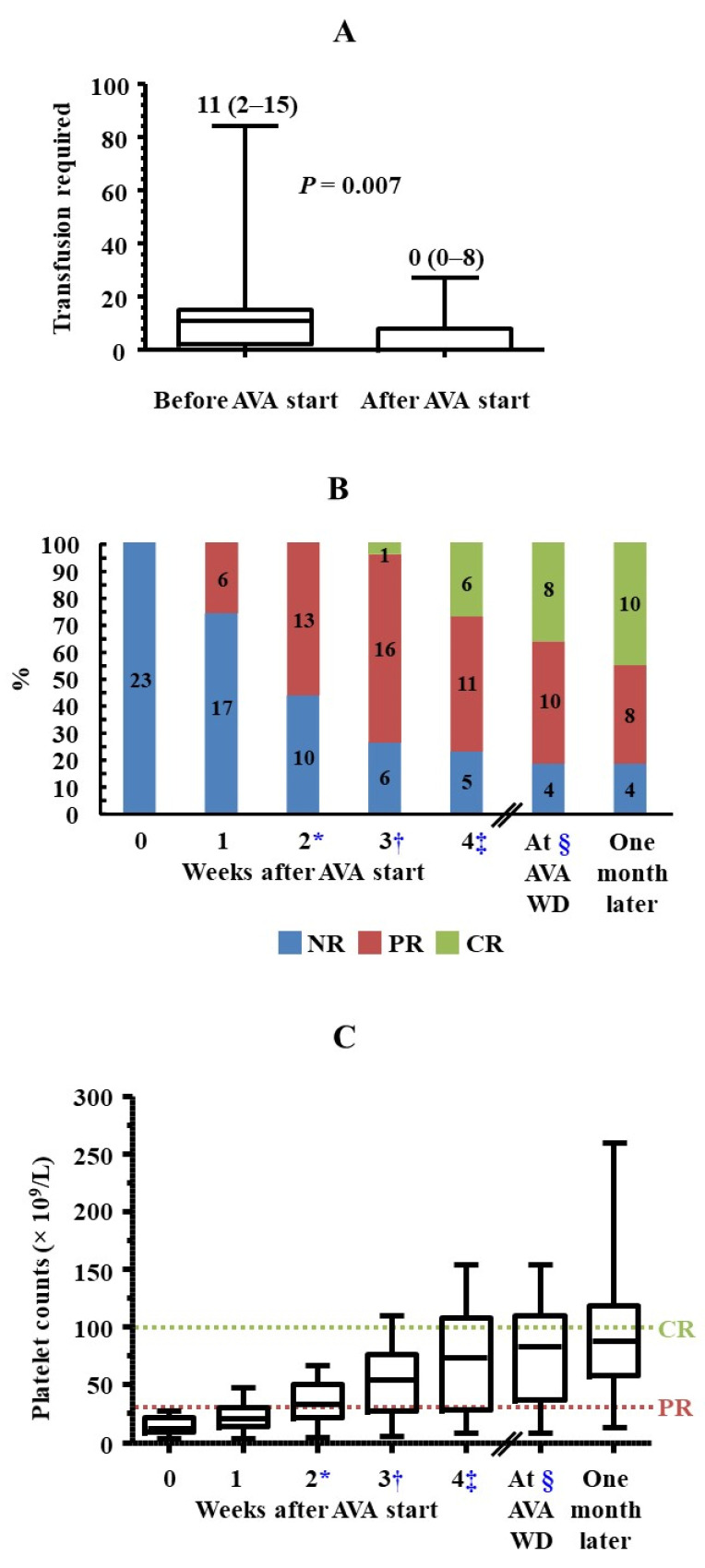
Transfusions required and platelet counts before and after starting AVA treatment in the overall cohort. (**A**) Whisker plots show the transfusions required after the onset of thrombocytopenia subsequent to antineoplastic therapies, according to whether patients were on AVA treatment or not. The median (IQR) number of transfusions is indicated for each period. (**B**) Response to AVA treatment and (**C**) whisker plots showing the platelet counts: on the one hand, immediately before AVA treatment and in the 4 weeks after treatment initiation; on the other hand, at the moment when AVA was suspended and one month after suspension. In (**C**), platelet count cut-off lines indicating PR (purple) and CR (green) are indicated. * One patient suspended treatment at this time point due to no response. † Three patients suspended treatment at this time point, due to no response or prolonged response in two and one cases, respectively, and one patient who had achieved PR was lost to follow-up due to transfer to another hospital. ‡ Nine patients suspended treatment at this time point, due to no response or prolonged response in one and eight cases, respectively. § In the overall cohort, AVA withdrawal took place at 5.0 (3.7–10.0) weeks after treatment initiation (median [IQR]).

Figure 2 summarises the main findings of the study. Thrombocytopenia, with platelet counts < 30 × 10^9^/L, occurred after a median (IQR) period of 15 (9–37) days following the start of chemotherapy. During the subsequent 3 weeks, when platelet counts remained persistently <30 × 10^9^/L, the median (IQR) number of platelet transfusions was 11 (2–15) (Figure 3A). Only 4 of 23 patients did not receive transfusions during this period, and none experienced bleeding episodes. Up to nine patients presented with haemorrhagic symptoms, most of them mild (cutaneous or gingival bleeding, epistaxis), although two grade 2 upper gastrointestinal bleeding episodes were also reported.

### 3.3. Efficacy of AVA

After 4 weeks of treatment with AVA, up to 18 of 23 (78.3%) patients responded to AVA, 6 (26.1%) of whom achieved CR. Six (26.1%), 13 (56.5%) and 16 (69.6%) patients achieved at least PR after one, two and three weeks of treatment, respectively. The median (IQR) treatment duration was 35 (25–72) days. AVA had been suspended in 13 cases by the end of the fourth week of treatment, with ineffectiveness or prolonged response being the cause of withdrawal in four and nine patients, respectively. One patient who had reached PR by the third week of treatment was then lost to follow-up. Therefore, nine patients continued with AVA beyond 28 days of treatment. The only patient who had not responded by the fourth week and did not suspend AVA achieved PR in the forthcoming days. At AVA withdrawal, 18 of 22 patients (81.8%) were in response, which was maintained one month after suspension in all of them (Figure 2, Figure 3B and Figure S1). Thus, all patients who responded to AVA maintained platelet counts ≥ 30 × 10^9^/L and did not report bleeding episodes until the study end, during a period of 53 (46–84) days since response was achieved.

In the overall cohort, median platelet counts exceeded 30 × 10^9^/L by the second week after the initiation of AVA therapy and increased progressively until the end of the study. One month after AVA withdrawal, all 18 patients who were in response maintained safe platelet counts, with levels ≥ 100 × 10^9^/L in 10 cases (Figure 3C and Figure S2).

The four patients who were refractory to AVA had undergone allo-HSCT and CAR-T cell therapy, two cases each. Three of them reported persistent cytopenias and the remaining one had developed acute liver graft-versus-host disease.

In the overall cohort, there was a significant inverse correlation between platelet counts immediately before the administration of the first dose of AVA and the number of platelet transfusions required: two-tailed Spearman’s rho = −0.725, *p* < 0.001. Nevertheless, the number of platelet transfusions required was remarkably and significantly lower once patients started treatment with AVA than in the previous period. The median (IQR) number of transfusions was 0 (0–8), and up to 13 patients never required this procedure, even though this phase lasted more than twice the time from the onset of thrombocytopenia to the first administration of AVA (Figure 2 and Figure 3A). When stratifying patients according to whether they underwent intensive chemotherapy (n = 12) or allo-HSCT (n = 8), the transfusion requirement since AVA initiation was significantly reduced in both groups. Finally, when transfusion requirement was studied in those patients who had platelet counts < 10 × 10^9^/L when AVA was started (n = 8), a smaller, non-statistically significant decrease was observed upon treatment use [13 (11–24) vs. 10 (2–14) transfusions before and after AVA initiation, respectively, *p* = 0.250]. In the overall cohort, no bleeding episodes were reported since AVA was started, except for an intracranial haemorrhage secondary to head trauma, which required 27 platelet transfusions to maintain a safe platelet count threshold.

Response to AVA was studied in two fragile subgroups, which consisted of patients with either platelet counts < 10 × 10^9^/L regardless of bleeding status or platelet counts between 10 × 10^9^/L and 30 × 10^9^/L with bleeding symptoms (Table 2). In both cases, response (PR or CR) was above 70% and was maintained until the end of the study. Nevertheless, patients with baseline platelet counts < 10 × 10^9^/L never reached CR. Conversely, none of those with baseline platelet counts between 10 × 10^9^/L and 30 × 10^9^/L required platelet transfusions.

Response to AVA was compared according to the procedure undergone by patients: intensive chemotherapy (n = 12) or allo-HSCT (n = 8). All patients in the first subgroup and 6 of 8 in the second responded to treatment. However, only 1 of 8 (12.5%) patients who underwent allo-HSCT reached CR, whereas 7 of 12 (58.3%) patients in the first subgroup achieved CR. Accordingly, the number of transfusions required once AVA therapy started was higher in allo-HSCT patients: 2 (0–10) vs. 0 (0–1) in patients receiving chemotherapy (Table S1).

### 3.4. AVA Dosing Throughout the Study

The initial AVA dose was established at 20 mg/day in all cases. In the 19 patients who responded to treatment, this dose was sufficient to achieve at least PR in 13 (68.4%) cases. Overall, AVA dose was increased to 40 mg/day in 11 (47.8%) patients. Nevertheless, the median dose was maintained at 20 mg/day throughout the study (Table S2). In the 8 patients who reached CR while on AVA treatment, this was achieved with the dose of 20 mg/day and 40 mg/day in 6 and 2 cases, respectively. In the case of patients who only reached PR, this was achieved with the dose of 20 mg/day and 40 mg/day in 4 and 6 cases, respectively.

Regarding AVA doses in the aforementioned fragile subgroups, only 1 of the 5 patients with baseline platelet counts < 10 × 10^9^/L who responded to treatment required an increase to 40 mg/day to achieve PR. The remaining responses were obtained with daily doses of 20 mg that were not increased throughout the study. In the 5 AVA responders who had baseline bleeding symptoms and baseline counts ≥ 10 × 10^9^/L, the AVA dose of 20 mg/day was sufficient to achieve PR in all patients and CR in 3 cases. Increasing the AVA dose to 40 mg/day allowed the remaining 2 patients to reach CR (Table 2).

### 3.5. Safety

AVA was well-tolerated by all patients, with no occurrence of serious TEAEs, bleeding complications, or thromboembolic events (TEVs). Only 5 (21.7%) patients experienced mild TEAEs. All 5 had asthenia, accompanied by nausea and vomiting in 2 cases. Twelve (52.2%) patients were managed without the need for hospital admission. Laboratory tests did not reveal major concerns. Haemoglobin levels and leukocyte counts were progressively increasing in the first 28 days after the start of AVA treatment, and no hepatobiliary damage was reported either (Table S3). Importantly, there was no need to delay anticancer procedures or reduce chemotherapy drug doses.

## 4. Discussion

Platelet transfusions remain the standard treatment for antineoplastic therapy-induced thrombocytopenia, but their benefit is transient, with inconsistent platelet recovery and risks of transfusion reactions or refractoriness. Consequently, transfusions are impractical for sustained platelet maintenance and are recommended only for active bleeding or severe thrombocytopenia, with platelet counts < 10 × 10^9^/L [7]. Thus, the current context of thrombocytopenia in clinical practice presents significant challenges, and alternative options are urgently required to fill this gap. The use of TPO-RAs for managing antineoplastic therapy-induced thrombocytopenia is supported by increasing evidence [25], and, as aforementioned, is encouraged by experts [11]. The National Comprehensive Cancer Network and the International Society for Thrombosis and Haemostasis endorse the off-label use of romiplostim, a subcutaneously injected TPO-RA, in selected patients with CIT [7,26]. However, the use of oral TPO-RAs in antineoplastic therapy-induced thrombocytopenia has yet to reach full consensus. Furthermore, experience in haematological malignancy is limited despite its high transfusion burden, and studies focused on AVA are almost unavailable in this field. Our findings are in line with those reported by others when AVA was used for managing thrombocytopenia secondary to allo-HSCT or chemotherapy in paediatric acute lymphoblastic leukaemia [19,20,21,22,23]. More than 80% of patients achieved platelet counts of at least ≥30 × 10^9^/L, half of them by the second week of treatment and most by the third week. Although the AVA dose was occasionally increased to 40 mg/day, the median dose was 20 mg/day throughout the study, and thus not higher than the initial dose recommended for managing ITP [27]. Interestingly, most patients who achieved CR were using the dose of 20 mg/day when reporting platelet counts ≥ 100 × 10^9^/L. However, more than half of patients who only reported PR, required a dose increase to 40 mg/day to achieve platelet counts ≥ 30 × 10^9^/L. This observation, together with the fact that a small fraction of refractory patients was also identified, suggests that there may be individual hallmarks conditioning response, which should be the matter of further investigation. Response was maintained during a median follow-up period of 53 (46–84) days, after which 45% of patients showed CR. Although response appeared slightly better in patients undergoing chemotherapy than in those receiving allo-HSCT, 75% of the latter had platelet counts ≥ 30 × 10^9^/L at the end of the study.

Importantly, the number of transfusions required since AVA initiation was markedly lower, both in the overall cohort and when patients were grouped according to whether they underwent intensive chemotherapy, allo-HSCT, or CAR-T cell therapy. More than half of the patients did not require platelet transfusion support throughout the treatment period, which means that AVA can be a useful tool to reduce transfusion burden and thereby mitigate transfusion-related risks while lowering healthcare costs [1,7]. Although results regarding transfusion requirement while on AVA treatment were less unequivocal in patients with platelet counts < 10 × 10^9^/L at the time the first dose of the TPO-RA was administered, the median number of transfusions in this subgroup was 10 over a median follow-up of 59 days of therapy. The exact number of transfusions required to maintain platelet counts above 10 × 10^9^/L depends on the severity of bone marrow suppression and other individual hallmarks, but it may increase up to 8 per chemotherapy cycle [28,29,30]. Therefore, AVA may also be useful for this purpose in severe conditions. Conversely, most patients with bleeding symptoms and platelet counts ≥ 10 × 10^9^/L at AVA initiation did not require platelet transfusions thereafter. It must be remarked that no bleeding episodes were reported in the overall cohort while on AVA and, when applicable, in the post-AVA period.

No safety concerns were reported in any patients while on AVA treatment. TEVs have been described occasionally in ITP patients treated with TPO-RAs [31]. No TEVs were documented in our cohort or in previous studies addressing the role of AVA in antineoplastic therapy-induced thrombocytopenia in haematological malignancy [19,20,21,22,23]. Nevertheless, studies covering longer treatment periods are required to assess long-term safety more accurately.

There is literature available describing the efficacy and safety profile of eltrombopag or romiplostim to manage antineoplastic therapy-induced thrombocytopenia in haematological malignancy [32,33,34,35,36,37,38]. Collectively, our results may suggest that AVA is not inferior to these TPO-RAs in restoring platelet counts in this scenario, and that its safety profile is not worse either. However, comprehensive meta-analyses or dedicated comparative studies evaluating the efficacy and safety profile of AVA versus ELT/ROMI are required to substantiate this hypothesis. A follow-on single-centre study comparing our previous experience with ELT/ROMI against AVA in this clinical setting is currently being planned.

Our study has limitations. The heterogeneity of haematological diseases and the size of the cohort preclude drawing robust conclusions, as well as focusing on chemotherapy procedures or specific pathologies. Seven patients who had undergone intensive chemotherapy were re-exposed again to cytostatic agents. However, the duration of the study does not allow the analysis of recurrent thrombocytopenia nor the assessment of the long-term effectiveness of AVA and, therefore, whether it can be useful to cover the whole chemotherapy regimen or for long-term management of thrombocytopenia in the context of allo-HSCT or CAR-T cell therapy, where prolonged thrombocytopenia has been described [39,40]. Platelet transfusions may have occasionally influenced platelet counts. Nevertheless, the number of transfusions, and therefore the risk of overestimating counts, was markedly higher in the pre-AVA period. Finally, the retrospective nature of the study precluded the evaluation of variables not routinely collected and potentially of interest, such as megakaryocyte count, platelet size dynamics, and markers of platelet maturity.

## 5. Conclusions

In summary, our study supports the notion that AVA may serve as a safe and effective treatment option for managing persistent antineoplastic therapy-induced thrombocytopenia in haematological patients. AVA appears to increase platelet counts and lessen the need for transfusions, potentially lowering bleeding risks, shortening hospital stays, and reducing costs. Additional research is needed to confirm these findings in specific conditions and to establish optimal treatment durations and dosing regimens.

## Figures and Tables

**Table 1:** Patient and disease characteristics in the overall cohort.

Characteristic	Value
Age at onset of thrombocytopenia, years, median (IQR)	64 (48–70)
Sex, male	14/23 (60.9)
Diagnosis	
Lymphoproliferative disorders	11/23 (47.8)
Chronic myeloproliferative neoplasms	5/23 (21.7)
Acute myeloid leukaemia	3/23 (13.0)
Multiple myeloma	2/23 (8.7)
Bone marrow aplasia	2/23 (8.7)
ECOG performance status, median (IQR) *	1 (0–2)
Secondary thrombocytopenia cause	
Intensive chemotherapy	12/23 (52.2)
Allogeneic haematopoietic stem cell transplantation	8/23 (34.8)
CAR-T cell therapy	3/23 (13.0)

Results are n/N (%), except where otherwise indicated. * Eastern Cooperative Oncology Group (ECOG) Performance Status reflects the patient’s functional ability and was assessed using a standardised clinical scale.

**Table 2:** Response to AVA and transfusion requirement in selected patient subgroups.

PLT Counts and Bleeding Status Before AVA	Transfusion After AVA Start	Best Response Achieved, n/N (%)			Response at Last Visit or AVA Suspension, n/N (%)			Follow-Up *
		NR	PR	CR	NR	PR	CR	
PLT counts < 10 × 10^9^/L (n = 8) †‡	10 (2–14)	2 §/7 (28.6)	5/7 (71.4)	0/7 (0)	2/7 (28.6)	5/7 (71.4)	0/7 (0)	59 (21–157) ¶
PLT counts ≥ 10 × 10^9^/L and bleeding (n = 6) #	0 (0–1)	1 **/6 (16.7)	0/6 (0)	5/6 (83.3)	1/6 (16.7)	0/6 (0)	5/6 (83.3)	25 (23–92) ††

Results are median (IQR) except where otherwise specified. * Days between the start of AVA treatment and either AVA withdrawal or last control visit. † Platelet counts immediately before AVA treatment were started, regardless of presence or absence of bleeding symptoms [median (IQR): 8.0 (4.3–9.6) × 10^9^/L]. ‡ One patient who had an intracranial haemorrhage secondary to a head trauma with no surgical options was not considered for calculations. § AVA suspended at 2 and 3 weeks after start, respectively. ¶ Five of seven (71.4%) patients suspended AVA. # Platelet counts immediately before AVA treatment was started [median (IQR): 21.5 (18.9–25.0) × 10^9^/L]. ** AVA suspended at 3 weeks after initiation. †† Five of six (83.3%) patients suspended AVA.

## Data Availability

The original contributions presented in this study are included in the article/Supplementary Material. Further inquiries can be directed to the corresponding author.

## Supplementary Materials

The following supporting information can be downloaded at https://www.mdpi.com/article/10.3390/jcm15052044/s1, Figure S1. Patient-by-patient summary of AVA dosing and treatment response throughout the study (A) AVA doses (mg/day, green) at treatment initiation, at weekly control visits during the first 4 weeks and, when applicable, at the final study visit, are shown. Responses to treatment (blue) at the corresponding time points are also indicated. When relevant, red circles denote the time at which AVA therapy was discontinued, except in the case of P19, where the red circle indicates loss to follow-up due to transfer to another hospital. “1st”, “2nd”, “3rd” and “4th” refer to the control visits in weeks 1, 2, 3 and 4 after the start of AVA therapy. “Last” refers to the last control visit, provided that AVA treatment had not been discontinued beforehand. Figure S2. Weekly monitoring of platelet counts. Each patient is represented with a coloured line, illustrating the evolution of platelet counts during weekly monitoring from the start of treatment until its interruption or last blood test. Table S1. Response to AVA and transfusion requirements according to anticancer procedure. Table S2. AVA dosing throughout the study. Table S3. Haemoglobin, leukocyte counts and hepatobiliary damage biomarkers during AVA treatment.

## Abbreviations

The following abbreviations are used in this manuscript:

Allo-HSCT	Allogeneic haematopoietic stem cell transplantation
ALT	Alanine aminotransferase
ALP	Alkaline phosphatase
AST	Aspartate aminotransferase
AVA	Avatrombopag
CAR-T	Chimeric antigen receptor T
CIT	Chemotherapy-induced thrombocytopenia
CR	Complete response
CT	Chemotherapy
ECOG	Eastern Cooperative Oncology Group
GGT	Gamma-glutamyl transferase
Hb	Haemoglobin
IQR	Interquartile range
ITP	Immune thrombocytopenia
mo	month
NR	No response
PC	Platelet counts
PLT	Platelets
PR	Partial response
RDI	Relative dose intensity
TEAEs	Treatment-emergent adverse events
TEVs	Thromboembolic events
TLC	Total leukocyte count
TPO-RAs	Thrombopoietin receptor agonists
WD	Withdrawal
wk	week
