# Supplementary material for: Avatrombopag Reduces Platelet Transfusion Requirement in Thrombocytopenia Subsequent to Antineoplastic Therapies in Haematological Patients: The Experience of a Tertiary Centre
Source: J Clin Med. 2026 Mar 7;15(5):2044. doi: 10.3390/jcm15052044 (PMC12986316; doi:10.3390/jcm15052044)
Supplement: Supplementary file 1 [file jcm-15-02044-s001.zip › jcm-4121224-supplementary.pdf]

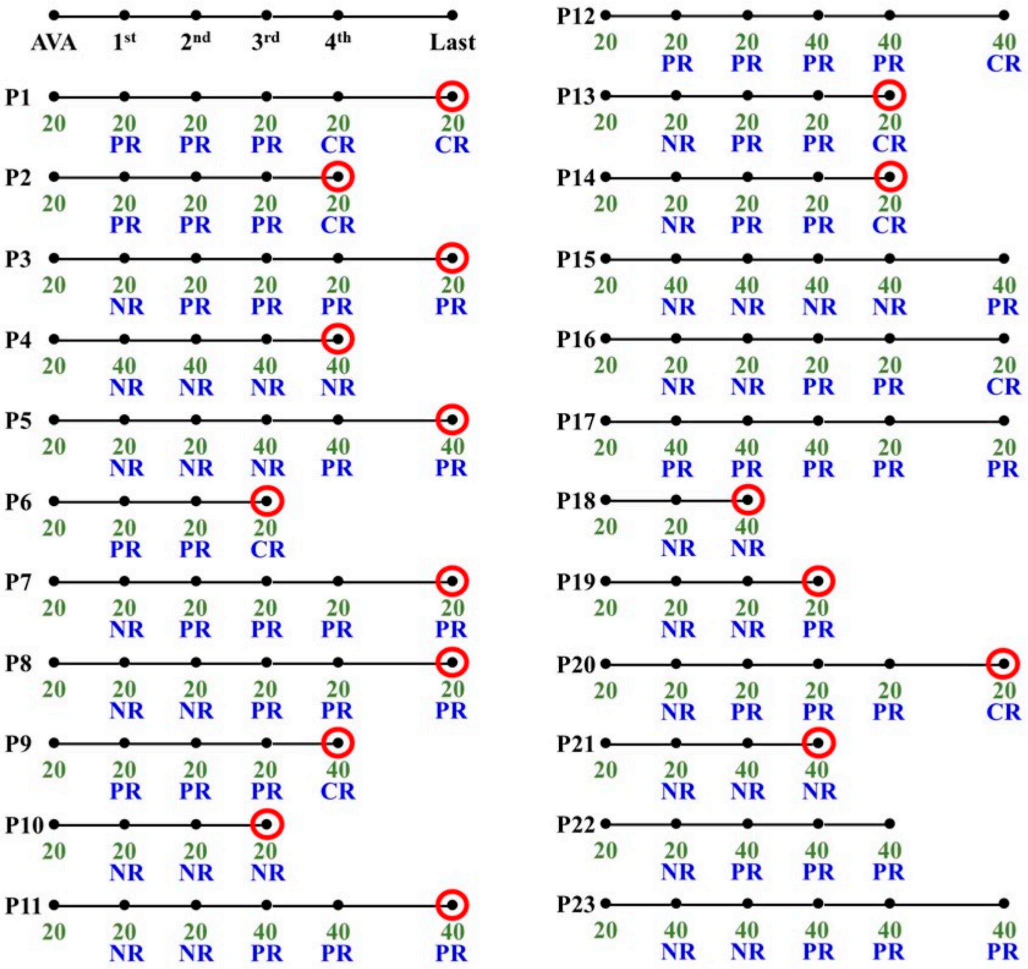

Figure S1. Patient-by-patient summary of AVA dosing and treatment response throughout the study (A) AVA doses (mg/day, green) at treatment initiation, at weekly control visits during the first 4 weeks and, when applicable, at the final study visit, are shown. Responses to treatment (blue) at the corresponding time points are also indicated. When relevant, red circles denote the time at which AVA therapy was discontinued, except in the case of P19, where the red circle indicates loss to follow-up due to transfer to another hospital. "1st", "2nd", "3rd" and "4th" refer to the control visits in weeks 1, 2, 3 and 4 after the start of AVA therapy. "Last" refers to the last control visit, provided that AVA treatment had not been discontinued beforehand.

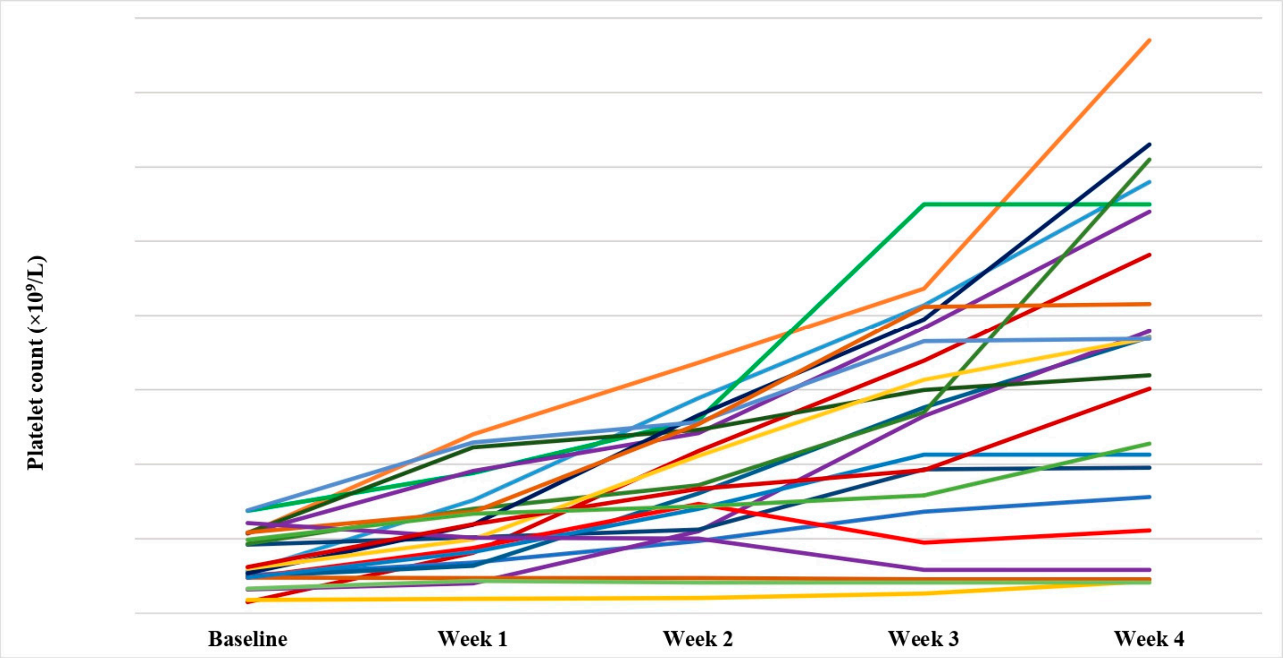

Figure S2. Weekly monitoring of platelet counts. Each patient is represented with a coloured line, illustrating the evolution of platelet counts during weekly monitoring from the start of treatment until its interruption or last blood test.

Table S1. Response to AVA and transfusion requirements according to anticancer procedure.

Type of Procedure	Best Response Achieved			*P**	Transfusion Required, Median (IQR)	*P*†
	**NR**	**PR**	**CR**	0.051		0.074
Intensive CT	0/12 (0)	5/12 (41.7)	7/12 (58.3)		0 (0–1)	
Allo-HSCT	2/8 (25.0)	5/8 (62.5)	1/8 (12.5)		2 (0–10)	

*Chi-square test. †Two-tailed Mann–Whitney U test.

Table S2. AVA dosing throughout the study.

Initial Dose (n = 23)	1st Week (n = 23)	2nd Week (n = 23)	3rd Week (n = 22)	4th Week (n = 18)
20 (20–20)	20 (20–40)	20 (20–40)	20 (20–40)	20 (20–40)

Results are expressed as mg/day, median (IQR).

Table S3. Haemoglobin, leukocyte counts and hepatobiliary damage biomarkers during AVA treatment.

Days on AVA	Hb (g/dL)	TLC (10^9/L)	AST (U/L)	ALT (U/L)	GGT (U/L)	ALP (U/L)
0 (start)	8.6 (7.4–10.4)	2.4 (1.7–3.6)	16.5 (12.7–32.0)	18.0 (11.0–33.0)	55.0 (23.0–107.0)	97.5 (85.5–173.0)
7	8.6 (7.6–10.0)	3.0 (2.0–5.6)	21.0 (14.0–27.0)	20.0 (13.0–39.0)	48.0 (23.0–88.0)	109.0 (75.0–157.0)
14	9.4 (7.7–10.6)	3.0 (2.1–5.8)	22.0 (17.0–29.0)	22.0 (12.0–35.0)	46.0 (28.0–97.0)	120.0 (80.0–152.0)
21	9.9 (8.4–11.2)	3.0 (2.1–5.6)	20.5 (14.7–26.5)	17.5 (12.0–27.0)	42.5 (23.0–74.2)	115.5 (81.5–157.0)
28	10.8 (9.4–11.6)*	4.8 (2.9–5.5)	24.0 (18.7–41.5)	19.5 (12.2–35.5)	40.0 (19.0–81.5)	115.5 (92.0–157.5)

For each variable, the Kruskal–Wallis test was applied. In case of statistical significance, the post hoc Dunn's test was used to compare values at baseline (day 0) vs. the other assessments. *$P < 0.05$.
